# MiR-591 functions as tumor suppressor in breast cancer by targeting TCF4 and inhibits Hippo-YAP/TAZ signaling pathway

**DOI:** 10.1186/s12935-019-0818-x

**Published:** 2019-04-24

**Authors:** Xin Huang, Fen Tang, Zeping Weng, Mengyao Zhou, Qing Zhang

**Affiliations:** 1 Department of Breast Surgery, The First Affiliated Hospital of Jinan University, 613 West Huangpu Road, Guangzhou, 510630 Guangdong People’s Republic of China; 2 Department of Pathology, The First Affiliated Hospital of Jinan University, Guangzhou, 510630 Guangdong People’s Republic of China; 3 Department of Experimental Center, The First Affiliated Hospital of Jinan University, Guangzhou, 510630 Guangdong People’s Republic of China

**Keywords:** Breast cancer, MicroRNAs, miR-591, TCF4, YAP1

## Abstract

**Background:**

MicroRNAs have been involved in regulating crucial biological function in some tumors. However, the clinical role and functional effects of miR-591 in breast cancer remain unknown.

**Methods:**

The expression of miR-591 was detected in breast cancer tissues and their paired normal tissues by qRT-PCR. Functional assays were performed to confirm the effects of miR-591 on the proliferation and invasion of breast cancer. Bioinformatics analysis, luciferase reporter assays, western blot and in vitro assays were used to confirm that TCF4 was a target gene of miR-591. Western blot analysis was carried out to analyze the relationship between miR-591 expression and YAP1 expression in breast cancer.

**Results:**

We found that miR-591 expression levels were significantly downregulated in breast cancer tissues compared to adjacent normal tumor tissues. Lower miR-591 expression notably related to lymph node metastasis and advanced TNM stage in patients with breast cancer. In vitro, cell proliferation and invasion were inhibited by transfection of miR-591 mimic in breast cancer cells, but were promoted by transfection of miR-591 inhibitor, compared to the controls. In vivo, we also found that miR-591 mimic significantly inhibited cell proliferation ability. Moreover, we identified that TCF4 was a direct target of miR-591 in breast cancer. TCF4 mediated the inhibiting effects of miR-591 on cell proliferation and invasion in breast cancer cells. In additional, we revealed that miR-591 overexpression significantly inhibited the Hippo-YAP/TAZ signaling pathway in breast cells by downregulated YAP1 expression in breast cells.

**Conclusion:**

Together, these results indicated that miR-591 is downregulated in breast cancer and could act as a potential target of breast cancer treatment.

## Background

Breast cancer is the most common invasive cancer in women worldwide [1]. About 252, 710 new cases are diagnosed and more than 40,000 women are likely to die from this disease [2]. In spite of the mortality rate of patients with breast cancer has been reduced due to the advances in diagnostic methods and therapeutic strategies [3]. However, patients with this disease present tumor metastasis at the late advance stage, leading to poor outcomes and higher mortality [3, 4]. Thus, to investigate new therapeutic strategies for breast cancer is needed.

MicroRNAs (MiRNAs), a series of small endogenous RNAs, are identified as crucial regulators in breast cancer development and progression [5]. MicroRNAs can regulate downstream gene expression by binding to the 3′-untranslated regions of their target mRNAs [6, 7]. For some microRNAs have been reported to act as regulators in breast cancer progression. MicroRNA-218 inhibits proliferation and invasion in ovarian cancer by targeting Runx2 [8]. MiR-139-5p modulates radiotherapy resistance in breast cancer by repressing multiple gene networks of DNA repair and ROS defense [9]. MiR-17-5p suppresses cell proliferation and invasion by targeting ETV1 in triple-negative breast cancer [10]. Moreover, more and more microRNAs were reported to participate in breast cancer biological function.

MiR-591 is identified as tumor suppressor in some tumors. A study showed that upregulation of miR-591 could confer paclitaxel resistance to ovarian cancer [11]. MiR-591 overexpression cellular growth, proliferation, and invasive capability in malignant pleural mesothelioma (MPM) [12]. In the study, we found that miR-591 expression was significantly downregulated in breast cancer tissues. In vitro, we revealed that miR-591 could inhibited cell proliferation and invasion by regulating TCF4 expression. In vivo, we also found that miR-591 mimic significantly inhibited cell proliferation ability. Moreover, we demonstrated that miR-591 overexpression inhibited the Hippo-YAP/TAZ signaling pathway in breast cells by downregulated YAP1 expression. Thus, these results indicated that miR-591 may function as a potential target of breast cancer treatment.

## Materials and methods

### Clinical tissue samples and cell lines

A total of 78 pairs of human breast cancer tissue samples and corresponding adjacent normal tissues were obtained from patients who underwent resection at Department of Breast Surgery, The First Affiliated Hospital of Jinan University between August 2014 and July 2016. All of the patients were female and the median age of the patients was 50 years (range from 28 to 76 years old). The study was approved by the Ethical Committee of The First Affiliated Hospital of Jinan University (the protocol number IACUC-20170602-01) and all of the patients provided written informed consent. Pathological classification and staging were based on the 2007 International Breast Cancer Typing Guidelines.

Four human BC cell lines MCF-7, MDA-MB-231, T-47D, SKBR3 and a human normal breast epithelial cell line MCF-10A were purchased from the Institute of Biochemistry and Cell Biology, Type Culture Collection of the Chinese Academy of Sciences (Shanghai, China). Cells were cultured in Dulbecco’s modified Eagle’s medium (DMEM) supplemented with 10% fetal bovine serum (FBS, Gibco; Thermo Fisher Scientific, Inc., Waltham, MA, USA) in a humidified 5% CO_2_ incubator at 37 °C.

### Cell transfection

Chemically synthesized miR-591 mimic, miR-591 inhibitor or miR-negative control (NC), pcDNA-vector and pcDNA-TCF4 were obtained from Guangzhou RiboBio Co., Ltd. (Guangzhou, China). Cells were transfected using Lipofectamine 2000 (Invitrogen; Thermo Fisher Scientific, Inc.), according to the manufacturer’s protocol.

### Quantitative reverse transcription polymerase chain reaction (QRT-PCR) analysis

Total RNA was extracted from tissues and cell lines using TRIzol^®^ (Invitrogen; Thermo Fisher Scientific, Inc.). The total RNA was reverse transcribed to cDNA using the Prime Script RT reagent kit (Takara Bio, Inc., Otsu, Japan) according to the manufacturer’s protocol. The qRT-PCR reaction was performed using the SYBR Premix Ex Taq (Takara Bio, Inc., Otsu, Japan) on an ABI 7500 Sequence Detection system (Applied Biosystems). The U6 snRNA was used as an internal control. The thermocycling conditions was as follow: 95 °C for 30 s, followed by 40 cycles of 95 °C for 5 s and 60 °C for 30 s, and one cycle of 95 °C for 15 s, 60 °C for 60 s and 95 °C for 15 s for dissociation. The relative mRNA expression was calculated using the 2^−∆∆Cq^ methods.

### Cell proliferation assays

Cell proliferation ability were assessed by CCK8 cell proliferation. Transfected cells (3 × 10^3^ cells/per well) were seeded in six-well plates. Cells were cultured for 0, 24, 48 and 72 h. Then, CCK8 solution (10 µl) was added into each well to incubate for 2 h at 37 °C, the plates were detected at indicated time (0, 24, 48 and 72 h) using a microplate reader at a wavelength of 450 nm.

### Transwell invasion assay

After cells were transfected with miR-591 mimic, miR-591 inhibitor or miR-NC for 24 h, cells were seeded in Matrigel-coated chambers (8 μm pore size, BD Bioscience, USA). 1 × 10^5^ MCF-7 cells or MDA-MB-231 cells were added in the upper chamber with serum-free medium. Medium containing 10% FBS was placed in the lower chambers to act as a chemoattractant. After 48 h, cells were fixed and then stained with 0.1% crystal violet. Cell invasing number was assessed at five fields under a light microscope (Ti; Nikon Corporation, Tokyo, Japan) at magnification of 200×.

### Dual luciferase reporter assay

Potential miR-591 binding sites were predicted using TargetScan (http://www.targetscan.org). The sequence of TCF4 with the wild-type or mutant 3′UTR seed region was synthesized and cloned into a pMIR-REPORT luciferase vector (Applied Biosystems; Thermo Fisher Scientific, Inc.). Then, The TCF4 with the wild-type or mutant 3′UTR pMIR-REPORT vector was co-transfected into MCF-7 cells with miR-591 mimic or miR-NC using Lipofectamine 2000 (Invitrogen; Thermo Fisher Scientific, Inc.). Firefly luciferase activity was detected at 48 h after transfection normalized against Renilla luciferase activity by using the Dual Luciferase Reporter Assay system (Promega Corporation, Madison, WI, USA) according to the manufacturer’s protocol.

### Western blotting analysis

Total protein was extracted from transfected cells by using a lysis buffer containing 150 mmol/l NaCl, 50 mmol/l Tris (pH 7.4), 1% Triton X-100, 1% sodium deoxycholate, 0.1% SDS, and protease inhibitor cocktail (Sigma-Aldrich; Merck KGaA, Darmstadt, Germany). Protein concentration was detected by using bicinchoninic acid assay kit (BCA) (Beyotime Institute of Biotechnology, Haimen, China). A total 30 µg protein was added on 10% polyacrylamide gel and transfer to a polyvinylidene (PVDF) membrane. The membranes were blocked with 5% skimmed milk (Sigma-Aldrich; Merck KGaA, Darmstadt, Germany) for 1 h. Then, the membranes were incubated with primary antibody against TCF4 (Abcam, Cambridge, UK), YAP1 (Santa Cruz Biotechnology, Inc.), LATS1 (Santa Cruz Biotechnology, Inc.) and GAPDH (Cell Signaling Technology Inc.) at 4 °C overnight. Subsequently, the membranes were incubated with the corresponding horseradish peroxidase (HRP)-conjugated secondary antibody (Epitomics; Abcam, Cambridge, UK) at room temperature. The membranes were detected by using an enhanced chemiluminescence detection kit (Pierce; Thermo Fisher Scientific, Inc.) and the GAPDH expression was used as an internal control. Each experiment was repeated three times.

### Tumorigenesis in nude mice

All animal experiments were conducted in accordance with current Chinese regulations and standards regarding the use of laboratory animals, and all animal procedures were approved by The First Affiliated Hospital of Jinan University Animal Care and Use Committee (the protocol number IACUC-20180101-01). 4-week-old BABL/c nude mice were purchased from the Center of Laboratory Animal Science of Guangdong (Guangzhou, China). Xenograft tumors were generated by subcutaneous injection of 1 × 10^6^ stable MCF-7 cells of miR-vector and miR-mimic on the hindlimbs. After 4 weeks, all mice were euthanized by dislocating the cervical spine. Tumor size was measured by a slide caliper (volume = length × width × height).

### Statistical analysis

All results are showed as the mean ± standard deviation (SD). Differences between groups were assessed using SPSS software (version 13.0; SPSS, Inc., Chicago, IL, USA). Data were analyzed with Student’s *t* test between two groups or one-way analysis of variance among more than two groups. Students-Newman-Keuls was performed to compare among more than two groups. P < 0.05 was considered to indicate a statistically significant difference.

## Results

### MiR-591 expression is downregulated in breast cancer tissues and cells

**Fig. 1:**
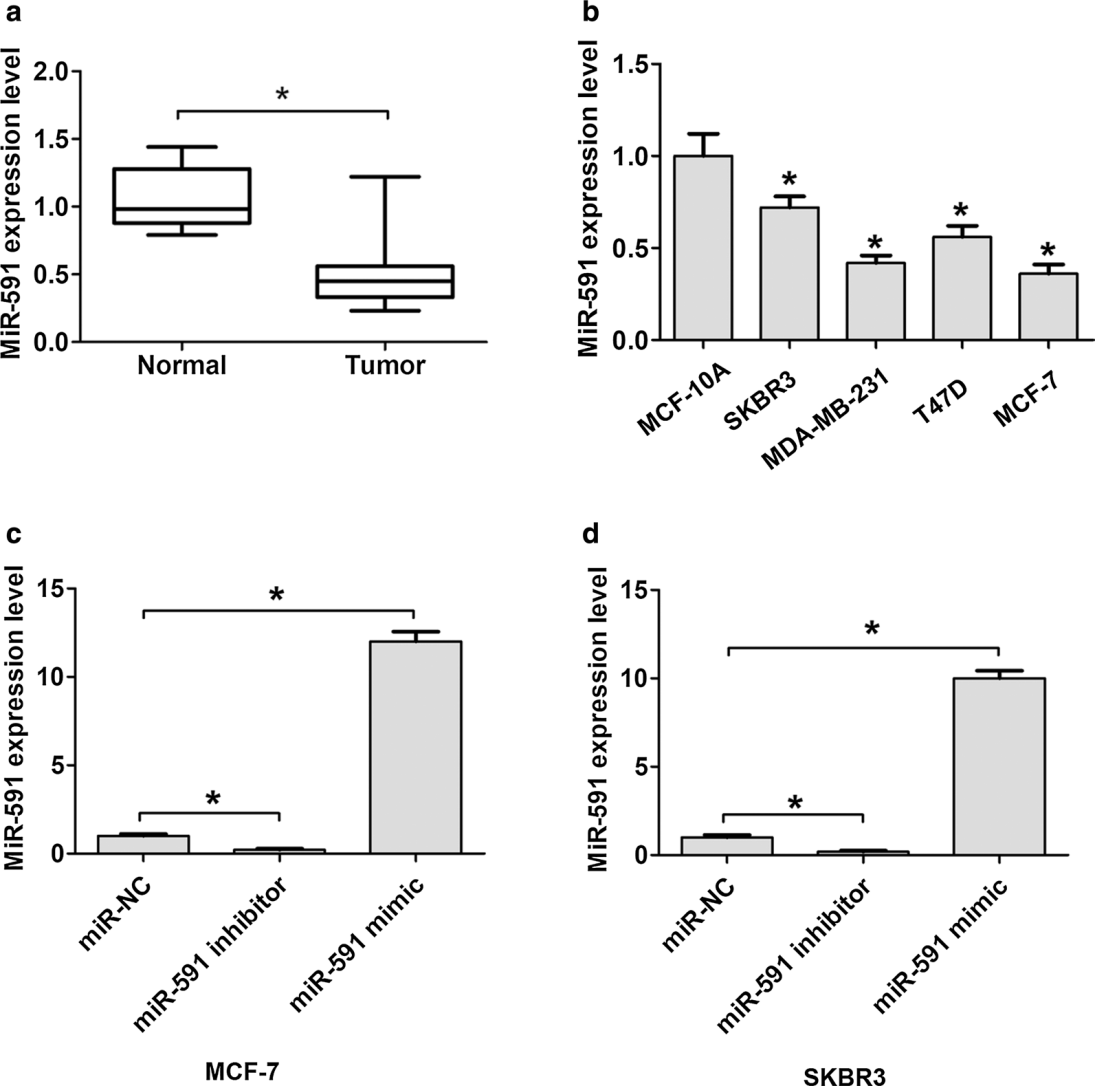
Decreased miR-591 expression in breast cancer tissues and cell lines. **a** miR-591 expression levels in breast cancer and adjacent normal tissues was detected by qRT-PCR. **b** The miR-591 expression levels were detected in MCF-10A, T-47D, MDA-MB-231 or MCF7 cell lines. **c** The miR-591 expression levels were detected in MCF-7 cells after cells were transfected with miR-591 mimic, miR-591 inhibitor or miR-NC. **d** The miR-591 expression levels were detected in SKBR3 cells after cells were transfected with miR-591 mimic, miR-591 inhibitor or miR-NC. All the data are expressed as the mean ± SD. *P < 0.05. *qRT-PCR* quantitative real-time PCR, *SD* standard deviation

To explore the clinical role in breast cancer, we examined the miR-591 expression in breast cancer tissues and adjacent normal tissues by qRT-PCR methods. The results observed that miR-591 expression is dramatically downregulated in breast cancer than that in adjacent normal tissues (Fig. 1a). The mean level of miR-591 expression in breast cancer tissues was used as a threshold to divide patients into two groups (lower and higher expression groups). Association between miR-591 expression and clinical data of patients with breast cancer was analyzed. Results showed that miR-591 expression significantly correlated with advanced TNM stage (P = 0.011) and lymph node metastasis (P = 0.005) of patients (Table 1).

**Table 1:** The association of clinicopathological factors with miR-591 expression in 78 breast cancer patients

Characteristics	Total	MiR-591 expression level		P-value
		Lower (n = 38)	Higher (n = 40)	
*Age (years)*				0.267
≤ 45	32	18	14	
> 45	46	20	26	
*Tumor size (cm)*				0.519
< 2	30	16	14	
> 2	48	22	26	
*PR status*				0.223
No	30	12	18	
Yes	48	26	22	
*TNM stage*				0.011*
I/II	50	19	31	
III	28	19	9	
*Lymph node metastasis*				0.005*
No	53	20	33	
Yes	25	18	7	
*Bone metastasis*				0.615
No	70	34	36	
Yes	8	4	4	
ER				0.972
No	45	22	23	
Yes	33	16	17	
*Histological grade*				0.262
G1/2	52	23	29	
G3	26	15	11	

*ER* estrogen receptor, *PR* progesterone receptor

* P < 0.05

### MiR-591 affects cell proliferation and invasion ability

**Fig. 2:**
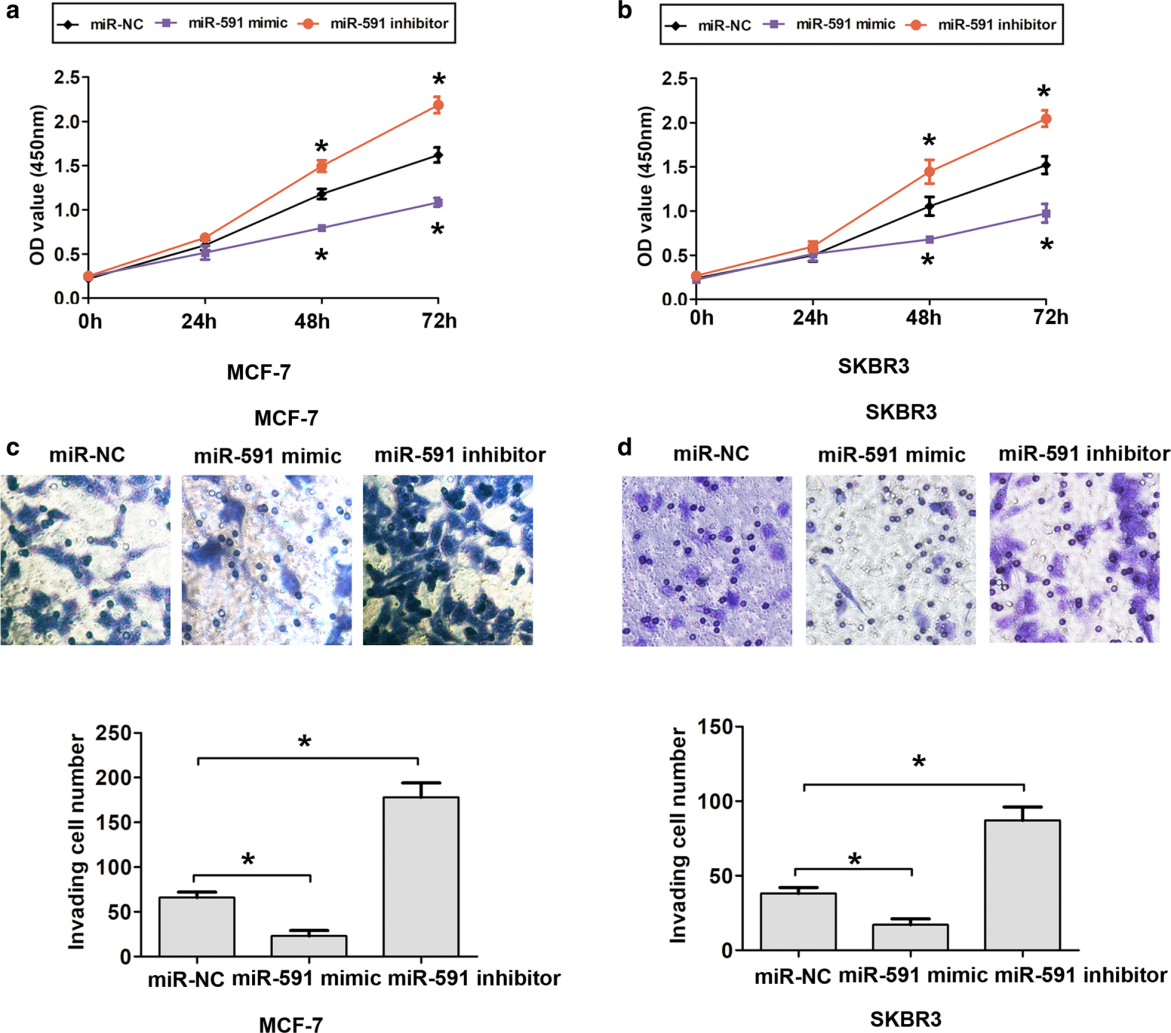
MiR-591 expression inhibits cell proliferation and invasion in breast cancer. **a** Cell proliferation ability was showed in MCF-7 cells in each transfected group as detected by Cell Counting Kit-8 assay. **b** Cell proliferation ability was showed in SKBR3 cells in each transfected group as detected by Cell Counting Kit-8 assay. **c** Cell invasion ability and invasive cell number was showed in MCF-7 cells in each transfected group as detected by tranwell assay. **d** Cell invasion ability and invasive cell number was showed in SKBR3 cells in each transfected group as detected by tranwell assay. All the data are expressed as the mean ± SD. *P < 0.05

Furthermore, we analyzed the expression of miR-591 in four human BC cell lines MCF-7, MDA-MB-231, T-47D, SKBR3 and a human normal breast epithelial cell line MCF-10A. The qRT-PCR assay results indicated that miR-591 expression was lower in breast cancer cells compared to MCF-10A cells (Fig. 1b). To evaluate the effects of miR-591 expression on cell proliferation and invasion, we performed gain and loss function assays. The results showed that miR-591 mimic transfected MCF-7 and MDA-MB-231 cells had a dramatic higher miR-591 expression, but miR-591 inhibitor transfected MCF-7 and MDA-MB-231 cells presented a lower miR-591 expression, compared to corresponding controls, respectively (Fig. 1c, d). Subsequently, the cell proliferation and invasion ability of MCF-7 and SKBR3 cells were determined using CCK8 assay and transwell assays. MCF-7 and SKBR3 cells transfected with miR-591 mimic were significantly reduced cell proliferation ability compared with the control cells at 48 h and 72 h, whereas cells transfected with the miR-591 inhibitor were significantly increased cell proliferation ability compared with the control cells (Fig. 2a, b). Furthermore, transfection of miR-591 mimic in MCF-7 and SKBR3 cells significantly inhibited cell invasion ability compared with the control cells at 48 h, whereas transfection of miR-591 inhibitor in MCF-7 and SKBR3 cells significantly enhancing cell invasion ability compared with the control cells (Fig. 2c, d). Thus, these results indicated that miR-591 inhibited cell proliferation and invasion ability of breast cancer.

### Upregulation of miR-591 suppresses the proliferation of breast cancer cells in vivo

**Fig. 3:**
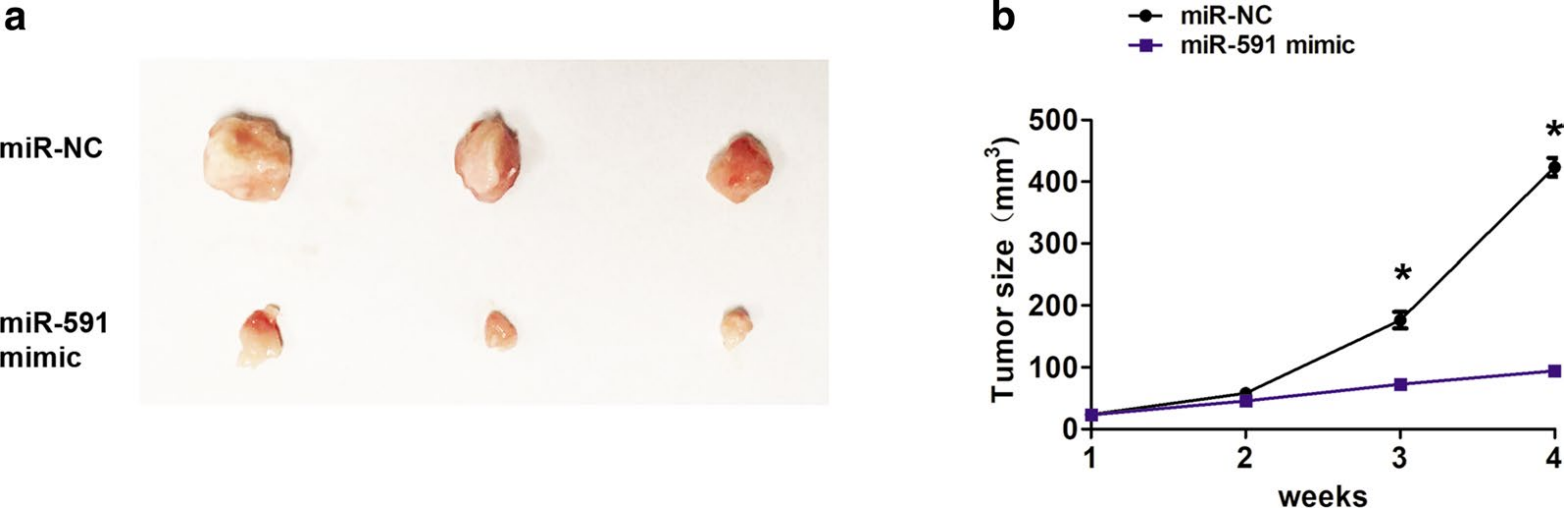
Upregulation of miR-591 suppresses the proliferation of breast cancer cells in vivo. **a** MiR-591 mimic group showed much smaller tumors volume than that in miR-NC group. **b** The tumor volume grown slower in miR-591 mimic group compared to that in miR-NC group. *P < 0.05

Furthermore, xenograft tumors were generated by subcutaneous injection of 1 × 10^6^ stable MCF-7 cells of miR-vector and miR-mimic on the hindlimbs. After 4 weeks, all mice were euthanized by dislocating the cervical spine. Tumor size was measured by a slide caliper (volume = length × width × height). Our results demonstrated that miR-591 mimic group showed much smaller tumors volume than that in miR-NC group (Fig. 3a). In addition, the tumor volume grown slower in miR-591 mimic group compared to that in miR-NC group (Fig. 3b). Thus, these results indicated that upregulation of miR-591 suppressed the proliferation of breast cancer cells in vivo.

### TCF4 is a direct target of miR-591 in breast cells

**Fig. 4:**
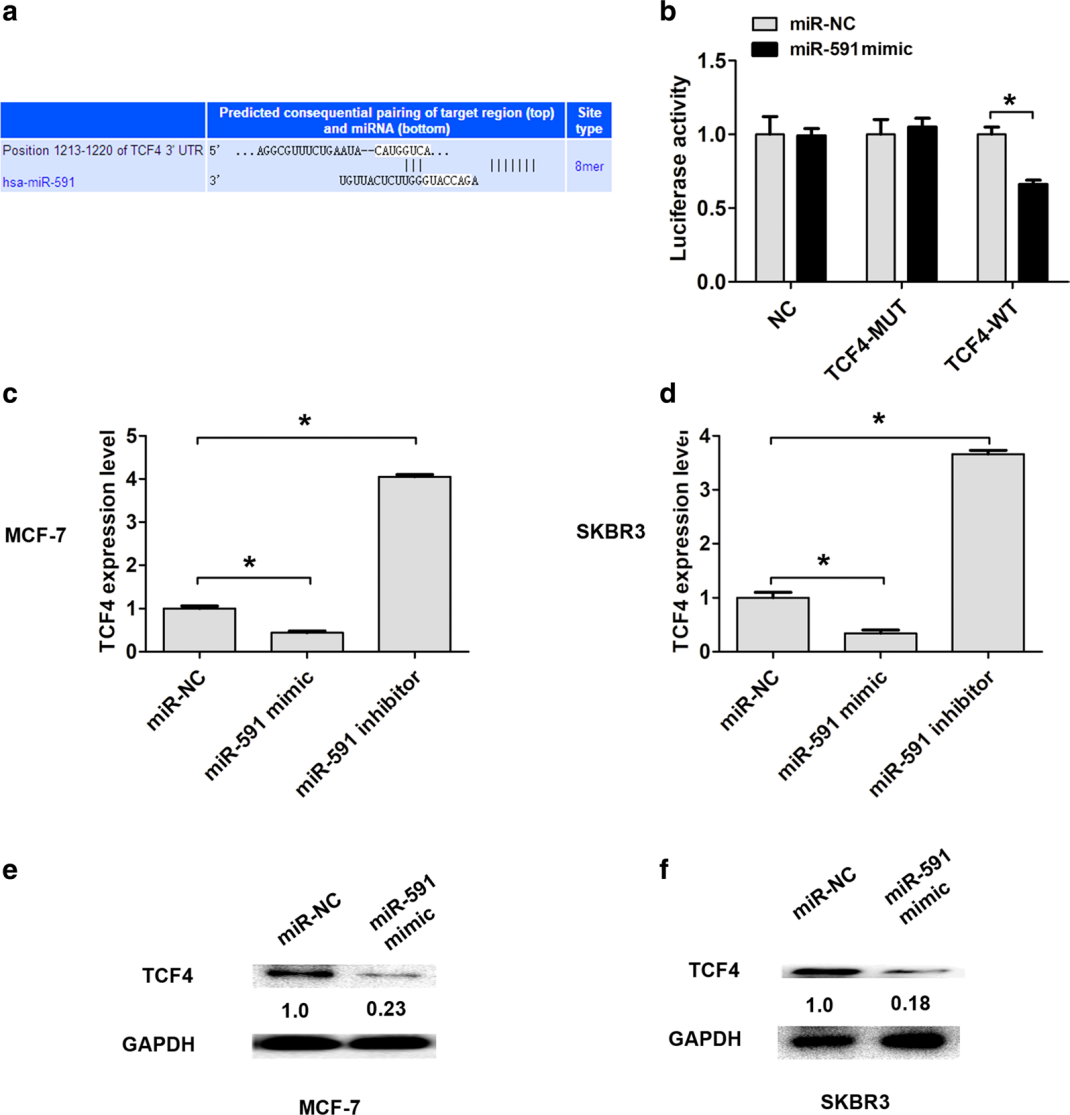
TCF4 is as target of miR-591 in breast cancer. **a** The miR-591-binding sites in the 3′-UTR of TCF4 was shown. **b** MCF-7 cells were co-transfected with pMIR-REPORT-TCF4-3′UTR WT or pMIR-REPORT-TCF4-3′UTR MUT, together with miR-591 mimic or miR-NC. Luciferase activity was measured at 48 h after transfection. **c**, **d** TCF4 mRNA expression in MCF-7 or SKBR3 cells transfected with miR-591 mimic, miR-591 inhibitor or miR-NC was measured using qRT-PCR. **e**, **f** TCF4 protein expression in MCF-7 or SKBR3 cells transfected with miR-591 mimic or miR-NC was measured using western blot analysis. All the data are expressed as the mean ± SD. *P < 0.05

By online databases (TargetScan and miRDB) predicted relative target of miR-591, we found that Transcription factor 4 (TCF4) was identified as a potential target of miR-591 (Fig. 4a). Furthermore, we co-transfected of TCF4-WT and miR-591 mimic in MCF-7 cells resulted in lower luciferase activity, while co-transfected of TCF4-MUT and miR-591 mimic showed no change in luciferase activity (Fig. 4b). Moreover, we detected the mRNA expression of TCF4 after upregulation of miR-591 or downregulation of miR-591 in MCF-7 or SKBR3 cells by qRT-PCR analysis. We found that transfection of miR-591 mimic in MCF-7 or SKBR3 cells significantly downregulated the mRNA expression of TCF4 (Fig. 4c, d). Transfection of MiR-591 inhibitor in MCF-7 cells resulted in upregulation of TCF4 expression (Fig. 4c, d). In additional, we also demonstrated that transfection of miR-591 mimic significantly downregulated the protein expression of TCF4 in MCF-7 or SKBR3 cells compared to the control groups (Fig. 4e, f). Thus, these results indicated that TCF4 was a direct target of miR-591 in breast cells.

### TCF4 up-regulation partially relieves miR-591 mediated suppression of cell proliferation and invasion in breast cancer

**Fig. 5:**
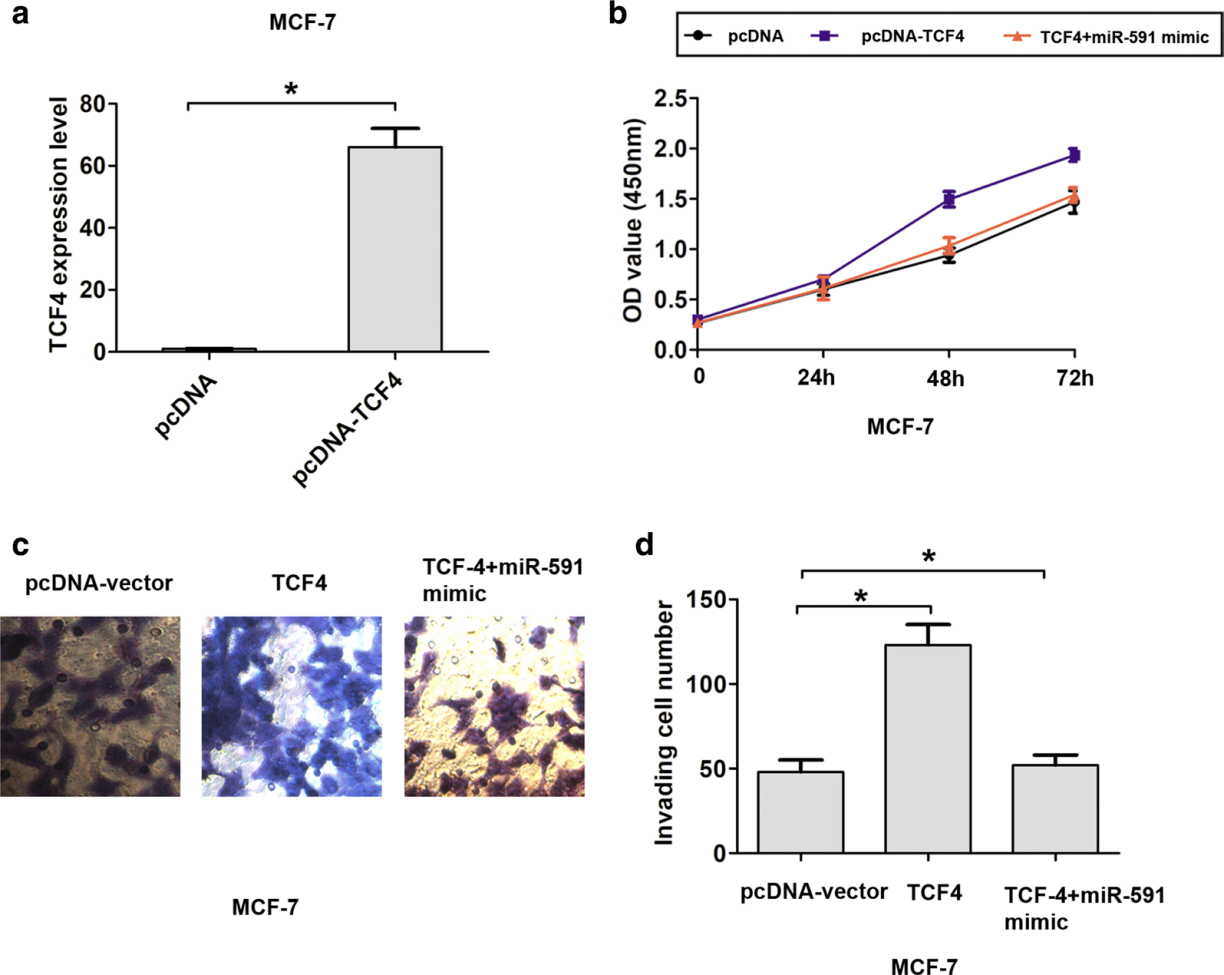
TCF4 up-regulation partially relieves miR-591 mediated suppression of cell proliferation and invasion in breast cancer. **a** TCF4 mRNA expression in MCF-7 cells transfected with pcDNA-vector or pcDNA-TCF4 was measured using qRT-PCR. **b** Cell proliferation ability was showed in MCF-7 cells in transfected group including pcDNA-vector, pcDNA-TCF4, TCF4 + miR-591 mimic as detected by Cell Counting Kit-8 assay. **c**, **d** Cell invasion ability was showed in MCF-7 cells in transfected group including pcDNA-vector, pcDNA-TCF4, TCF4 + miR-591 mimic as detected by transwell assay. All the data are expressed as the mean ± SD. *P < 0.05

To determine whether miR-591 suppresses cancer cell proliferation and invasion by regulating TCF4. We constructed TCF4 over-expressing vector and transfected into MCF-7 cells. Over-expression of TCF4 was confirmed by qRT-PCR analysis (Fig. 5a). The CCK8 cell proliferation and transwell assays showed that miR-591-mediated suppression of cell proliferation and invasion was partially rescued by overexpressed TCF4 in MCF-7 cells (Fig. 5b, d). Together, these data indicate that miR-591 suppresses cell proliferation and invasion by down-regulating TCF4.

### MiR-591 inhibits Hippo-YAP signaling pathway in breast cancer

**Fig. 6:**
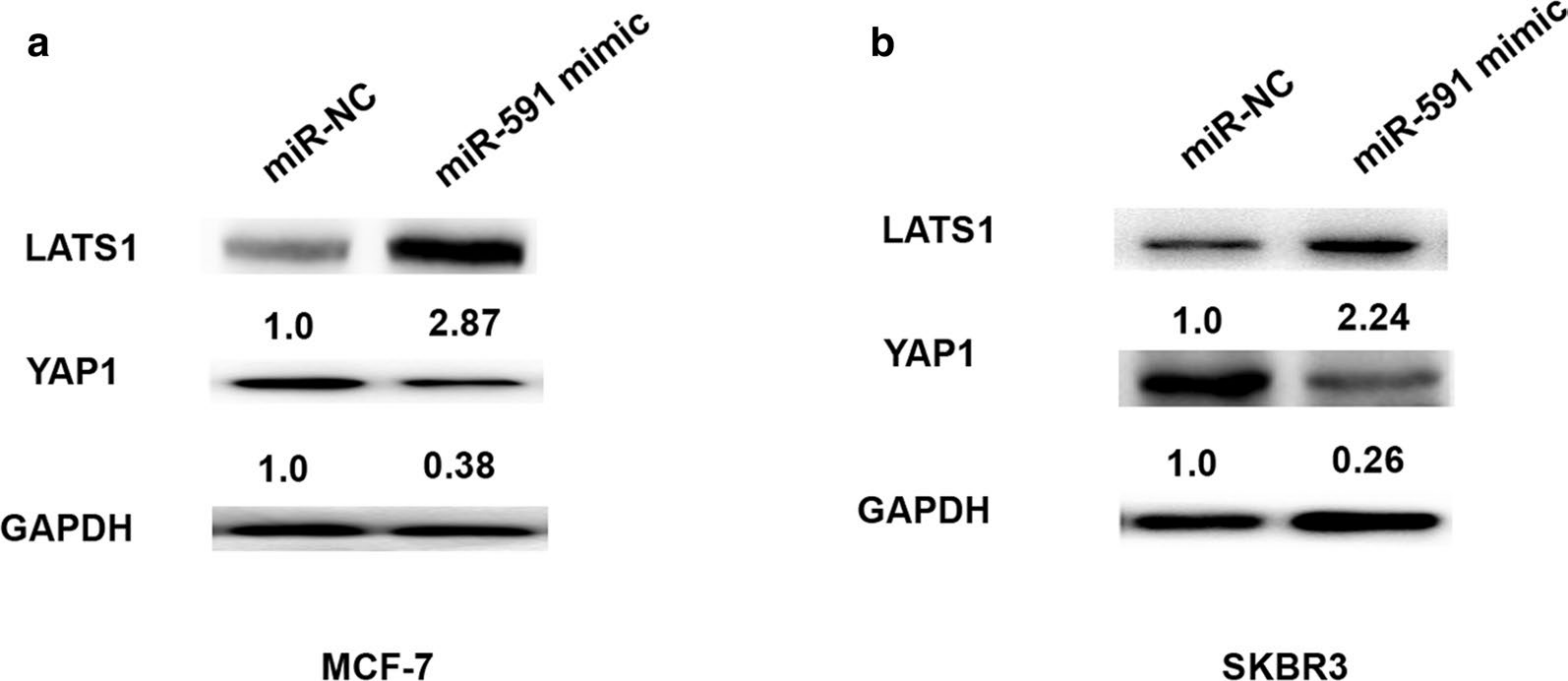
MiR-591 inhibits Hippo-YAP signaling pathway in breast cancer. **a** LATS1 and YAP1 protein expression in MCF-7 cells transfected with miR-591 mimic or miR-NC was measured using western blot analysis. **b** LATS1 and YAP1 protein expression in SKBR3 cells transfected with miR-591 mimic or miR-NC was measured using western blot analysis

Hippo-YAP signaling pathway has been reported to involved in tumor progression in breast cancer [13]. We detected the YAP expression by upregulation of miR-591 in MCF-7 and SKBR3 cells. The western blot analysis showed that YAP1 expression was downregulated after miR-591 upregulation in MCF-7 or SKBR3 cells compared to the control groups (Fig. 6a, b). However, LAST1 expression was upregulated after miR-591 upregulation in MCF-7 or SKBR3 cells compared to the control groups (Fig. 6a, b). Thus, these results indicated that miR-591 inhibited Hippo-YAP signaling pathway in breast cancer.

## Discussion

Breast cancer is a heterogeneous disease that includes various subtypes, with different biological behavior and clinical outcome. miRNAs have been found to be participated in breast cancer progression. MiR-591 have important roles in several tumors such, as miR-591 confers PTX resistance to ovarian cancer cells and modulation of MiR-591 resensitises PTX-resistant cancer cells by targeting ZEB1 [11]. However, the clinical role and functional effects of miR-591 in breast cancer remains unknown.

In the present study, we have ascertained novel molecular mechanisms miR-591 inhibited cell proliferation and invasion in breast cancer. We found that miR-519 expression was low in breast cancer tissues and cells. Lower miR-591 expression notably related to lymph node metastasis and TNM stage. Bone metastasis is frequent in breast cancer patients, in the study, the clinical analysis results showed no association between miR-519 expression and bone metastasis. We speculated the cases number may be inadequate and needed to extend the cases in the further. Moreover, the cell lines use in the study is not association with osteotropism, we did evaluate the expression of miR-591 is lower in several breast cancer cell lines. In vitro, cell proliferation and invasion were inhibited by transfection of miR-591 mimic in breast cancer. In vivo, our results also indicated that upregulation of miR-591 suppressed the proliferation of breast cancer cells in vivo. Moreover, we identified that TCF4 was a target of miR-591 in breast cancer. TCF4 play crucial functional role in breast cancer. The TCF4/β-catenin pathway and chromatin structure cooperate to regulate d-glucuronyl C5-epimerase expression in breast cancer [14]. MicroRNA-100 suppresses the migration and invasion of breast cancer cells by targeting FZD-8 and inhibiting T-cell factor-4 (TCF-4) expression [15]. By upregulating the TCF4 expression, we demonstrated that TCF4 mediated the inhibiting effects of miR-591 expression on cell proliferation and invasion in breast cancer cells.

Besides, we demonstrated that miR-591 overexpression inhibited the Hippo-YAP/TAZ signaling pathway in breast cells by downregulated YAP1 expression. Studies found that the Hippo-YAP/TAZ pathway mediates geranylgeranylation signaling in breast cancer progression [16]. Notch3 inhibits epithelial-mesenchymal transition by activating Kibra-mediated Hippo/YAP signaling in breast cancer epithelial cells [13]. Dual inhibition of WNT and Yes-associated protein signaling retards the growth of triple-negative breast cancer in both mesenchymal and epithelial states [17]. Thus, these results indicated that upregulation of miR-591 could inhibit the TCF4 and Hippo-YAP signaling, which may function as a potential target of breast cancer treatment.

## Conclusion

In conclusion, our results indicated that miR-591 expression was downregulated in breast cancer tissues and cells. Furthermore, we demonstrated that miR-591 could regulate cell proliferation and invasion by targeting TCF4 and inhibited the TCF4 and Hippo-YAP signaling, which may function as a potential target of breast cancer treatment.

## Abbreviations

MiRNAs: microRNA
DMEM: Dulbecco’s modified Eagle’s medium
FBS: fetal bovine serum
TCF4: transcription factor 4
